# Hardness-Dependent Water Quality Criteria for Protection of Freshwater Aquatic Organisms for Silver in China

**DOI:** 10.3390/ijerph19106067

**Published:** 2022-05-17

**Authors:** Qi Jin, Chenglian Feng, Peng Xia, Yingchen Bai

**Affiliations:** 1 State Key Laboratory of Environmental Criteria and Risk Assessment, Chinese Research Academy of Environmental Sciences, Beijing 100012, China; jinqi191@mails.ucas.ac.cn; 2 School of Environmental and Safety Engineering, Changzhou University, Changzhou 213164, China; 164380234@smail.cczu.edu.cn

**Keywords:** silver, water hardness, water quality criteria, species sensitivity distribution

## Abstract

Silver is toxic to freshwater aquatic organisms and aquatic ecosystems, and it is necessary to develop regional water quality criteria (WQC) for silver to protect the freshwater aquatic organisms in China. The toxicity database of silver for freshwater aquatic organisms involved 121 acute toxicity values for 35 species (6 phyla and 27 families) and 15 chronic toxicity values for 4 species (2 phyla and 4 families). Teleost fish showed the most sensitivity to silver after both short-term and long-term exposure. Significant correlations between the natural logarithms of hardness and the natural logarithms of acute silver toxicity were found for *Daphnia magna*, *Oncorhynchus mykiss*, and *Pimephales promelas*. The criterion maximum concentration (CMC) was calculated by the species sensitivity distribution method with sigmoid as the best fitting model (Adj *R*^2^ 0.9797), and the criterion continuous concentration (CCC) was obtained by the acute-to-chronic ratio method. The CMC and CCC of silver were $e^{1.58\ln\left(H_{{\mathrm{CaCO}}_{3}}\right)-8.68}$, and $e^{1.58\ln\left(H_{{\mathrm{CaCO}}_{3}}\right)-10.28}$ respectively, in China, with water hardness ($H_{{\mathrm{CaCO}}_{3}}$, mg/L) as an independent variable. This research can provide a basis and reference for the management of silver to protect freshwater aquatic organisms in China.

## 1. Introduction

Due to its excellent malleability, electrical and thermal conductivity, photosensitivity, ductility, and antimicrobial properties [1,2], silver, as an essential heavy metal, has been widely used in different industrial applications such as photographic manufacturing, electrical contacts, conductors, batteries, medical applications, catalysts, nanotechnology, etc., [3,4]. As a result of the large-scale industrial use of silver, silver ions inevitably enter the water during all stages of the product’s life cycle, including production, transport, storage, usage, and disposal [5]. The silver ion is an environmental and industrial pollutant with high acute and chronic toxicity to freshwater aquatic organisms [6,7]. For example, fish gills are respiratory organs with functions including gas exchange, ion regulation, filter feeding, acid–base balance, ammonia excretion, etc., [8]. Many researchers have documented that exposure to silver ions could cause lethal toxicity in fish (*Cyprinus carpio*, *Oncorhynchus mykiss*, etc.) due to gill damage and to fleas (*Daphnia magna*) due to inhibition of whole-body Na^+^ and K^+^-dependent adenosine triphosphatase, which is involved in ionoregulatory imbalance [9,10,11,12,13]. Due to its toxicity and environmental risk, silver is classified as a hazardous substance for freshwater aquatic organisms in many countries [6,14,15]. Water quality criteria (WQC) for the protection of freshwater aquatic organisms are the maximum concentrations of pollutants allowed in water that serve as the threshold for the health and safety of freshwater aquatic organisms [16,17,18]. WQC, as the scientific foundation of water quality standards, plays an important role in environmental management [19,20,21,22]. The WQC for silver have been investigated and promulgated for the protection of freshwater aquatic organisms in many countries such as the United States, Canada, Australia, and New Zealand [23].

Most water quality standards in China have referred to the WQC of the developed countries; however, the effectiveness, fitness, and scientific accuracy of WQC have been questioned, as they pertain to protecting the bio-environmental system in China. For example, it has been suggested that the WQC for the salmonidae and cyprinidae families be derived from the guidelines issued by the United States Environmental Protection Agency (USEPA) and the Ministry of Ecology and Environment of the People’s Republic of China (MEEC), respectively, due to the differences in freshwater biota across different countries [24,25]. To date, technical guidelines and three WQC documents on cadmium, ammonia, and phenol have been issued by the MEEC [26,27,28,29]. The three main sources of silver are reported to include natural sources, industrial discharge, and domestic discharge in China [14]. For example, the electroplating industry discharges the greatest amount of wastewater containing silver in China [30,31]. Meanwhile, the possible applications of antibacterial agents and nanoparticles containing silver are other sources of silver ions in freshwater in China. However, no WQC for silver to protect freshwater aquatic organisms have been issued in China, which is not conducive to managing the silver in freshwater scientifically and effectively.

The species sensitivity distribution (SSD) method is a powerful and common method for calculating the WQC and has been recommended for deriving the WQC for pollutants all over the world [15,25,32,33]. The SSD method is based on the assumption that the species being tested is representative of all the species in the ecosystem as far as sensitivity is concerned. It has been shown that the SSD method has the advantages of being supported by statistical theory and being able to use a full array of toxicological data [34]. Therefore, the SSD method might be an effective tool to apply to derive the WQC for silver to protect freshwater aquatic organisms in China. In addition, the toxicity of silver may be affected by water quality parameters such as hardness [6,35]. For instance, a decrease in the silver accumulation and an increase in the cell density of *Raphidocelis subcapitata* have been verified to coincide with increasing water hardness after exposure for seven days [35]. Significantly positive relationships have also been documented by the USEPA between the hardness of experimental water and the acute toxicity of silver ions [6]. Therefore, it is necessary to derive the hardness-dependent WQC for silver.

The purposes of this work were (1) to compare the different sensitivities between species by compiling a toxicity database of silver for freshwater aquatic organisms, (2) to identify the quantitative correlation between the toxicity of silver and the hardness of the tested water, and (3) to derive WQC for silver to protect freshwater aquatic organisms in China.

## 2. Materials and Methods

### 2.1. Toxicity Data Collection and Selection

To derive the WQC for silver, freshwater aquatic organisms were selected based on native freshwater aquatic organisms, widely distributed common international species, and alien species distributed widely in the surface waters of China. The WQC included the criterion maximum concentration (CMC) and the criterion continuous concentration (CCC), which aimed to provide an appropriate extent of protection to freshwater aquatic organisms under acute and chronic toxicity, respectively. For the measurement endpoints, the acute toxicity endpoints included the median lethal concentration (LC_50_) and the median effect concentration (EC_50_). The exposure time for acute toxicity was 24–96 h, which was preferred to be 96 h for fish, 48 h for Daphnia, and 24–48 h for algae. The endpoints for the chronic toxicity included the no observed effect concentration (NOEC), the lowest observed effect concentration (LOEC), and the maximum acceptable toxicant (MATC). The exposure time of chronic toxicity was at least 21 days or across at least one generation for freshwater aquatic organisms. For example, the exposure time was at least four days for the chronic toxicity of algae, due to the rapid cell division rate [36]. If the LOEC and NOEC were given under the same experimental conditions, the MATC was calculated as the geometric mean of the NOEC and LOEC and applied to derive the WQC for silver ions.

The acute toxicity values (ATVs) and chronic toxicity values (CTVs) of silver for the freshwater aquatic organisms were collected from both databases from the literature and the toxicity database. The retrieval strategy was “TI = (Silver ion OR Ag) AND TS = (toxicity OR LC_50_ OR EC_50_ OR NOEC OR LOEC OR MATC)” for the literature, which was selected from the China Knowledge Resource Integrated Database (http://www.cnki.net/ (accessed on 31 December 2021)), Elsevier (http://www.sciencedirect.com (accessed on 31 December 2021)), and Web of Science (http://www.webofscience.com (accessed on 31 December 2021)). For the toxicity database of the ECOTOX database (http://cfpub.epa.gov/ecotox (accessed on 10 January 2022)), the retrieval strategy was “Chemicals = (silver) and Effects = (all) and Endpoints = (LC_50_ AND EC_50_ AND NOEC AND LOEC AND MATC) and Species = (both animals and plants) and Test condition = (fresh water)”. However, ATVs that did not report the hardness of the test waters were excluded due to the possible influence of hardness on the acute toxicity of silver for freshwater aquatic organisms.

The acute and chronic toxicity database should cover at least three phyla and eight families of freshwater aquatic organisms to protect the whole freshwater ecosystem and to ensure the stability of SSD derivation, which contained a cyprinid teleost fish, a non-cyprinid teleost fish, a planktonic species, a benthonic species, and a freshwater plant.

### 2.2. Hardness Adjustment of ATVs

The relationship between the water quality characteristics and the toxicity should be taken into account using an analysis of covariance when enough data are available to prove the relationship between two or more species [24,25]. The hardness adjustment was used as an analysis of covariance to estimate the effect of hardness in tested waters on the acute toxicity to silver of freshwater aquatic organisms. In order to enhance the reliability of the toxicity data, the ATVs for a species should cover a broad enough range of the water hardness that the highest hardness is at least three times the lowest, and at least 90 mg/L higher than the lowest hardness calculated with CaCO_3_.

The analysis of covariance evaluated the relationship between the natural logarithms of hardness and the natural logarithms of ATVs of the selected species. The species mean toxicity values included the species mean acute toxicity values (SMAVs) and the species chronic toxicity values (SMCVs), which were calculated as the geometric mean of the available ATVs and CTVs for each species individually. For a species, the available ATVs were divided by the SMAVs of each species, as normalized ATVs. The geometric mean of the hardness values for each species was calculated individually. For each species, the available hardness values divided by the geometric mean of each species were then normalized for hardness.

We performed a least-squares regression on the normalized ATVs corresponding to the normalized hardness to obtain the pooled slope (*K*_pooled_). The adjustment of the ATVs of silver for freshwater aquatic organisms based on the *K*_pooled_ is shown in Equation (1):

(1) $${\mathrm{ATV}}_{\mathrm{adj}}=e^{K_{\mathrm{pooled}}\times \ln\left(H_{{\mathrm{CaCO}}_{3}}\right)+\ln\left({\mathrm{ATV}}_{\mathrm{org}}\right)-K_{\mathrm{pooled}}\times \ln\left(H_{\mathrm{org}}\right)}$$

where ATV_org_ is the original ATV, *H*_org_ is the original water hardness, $H_{{\mathrm{CaCO}}_{3}}$ is the adjusted water hardness, and ATV_adj_ is the ATV adjusted to the water hardness.

### 2.3. Statistical Analysis and the CMC Derivation by SSD Method

The SSD method was used to derive the CMC of silver to protect the freshwater aquatic organisms. The SMAV_adj_ is the geometric mean of the ATVs_adj_ of silver adjusted to the water hardness for a species, as shown in Equation (2):

(2) $${\mathrm{SMAV}}_{\mathrm{adj}}=\sqrt[{N}]{{\mathrm{ATV}}_{\mathrm{adj},1}\times {\mathrm{ATV}}_{\mathrm{adj},2}\times \cdots \times {\mathrm{ATV}}_{\mathrm{adj},N}},$$

where *N* is the total number of ATVs for a species.

Then, the SMAVs_adj_ for all species were arranged in sequence as rank *R* from smallest (*R* = 1) to largest (*R* = *N*), and the cumulative probability (*P*) of the species was calculated using Equation (3):

(3) $$P=\frac{R}{N+1}\;$$

The SSD curves were created using five different models, including the sigmoid model, the logarithm model, the Lorentzian model, the Gompertz model, and the exponential growth model, to derive the CMC and used the natural logarithms of SMAVs_adj_ as the independent variable and the cumulative probability of the species as the dependent variable. The best model based on the adjusted coefficient of determination, *R*^2^ (Adj *R*^2^), was applied to predict the hazardous concentrations for 5% of species (HC_5_) to protect the other 95% of the species. The CMC of the WQC was defined as the acute HC_5_ value divided by an assessment factor, which ranged from 2–5 based on the quantity and quality of the ATVs [37].

### 2.4. The CCC Derivation by the Acute-to-Chronic Ratio (ACR) Method

The ACR was calculated as a ratio of ATVs to CTVs under the same experimental conditions. The species acute-to-chronic ratio (SACR) was calculated as the geometric mean of the ACR for certain species. The final acute-to-chronic ratio (FACR) was the geometric mean of the SACR for all species. Finally, the CCC was calculated by dividing the CMC by the FACR. Origin 8.0 (OriginLab, Northampton, MA, USA) and Sigmaplot 14.0 (Systat Software, Inc., San Jose, CA, USA) were used for data analysis for both the CMC and CCC.

## 3. Results and Discussion

### 3.1. The Database of ATVs and CTVs of Silver to Freshwater Aquatic Organisms

The collected published acute toxicity database of silver for Chinese freshwater aquatic organisms from the literature and toxicity database involved 121 ATVs for 35 species (6 phyla and 27 families), shown in Table 1 and Table S1 with the corresponding water hardness. The ATVs for silver ranged from 0.14 μg/L to 4500 μg/L, while the water hardness ranged from 11.3 mg/L to 255 mg/L as CaCO_3_. For acute toxicity, there was a freshwater plant; 13 invertebrate species including 9 planktonic species and 10 benthic species; and 15 vertebrate species including 3 cyprinid teleost fishes, 10 non-cyprinid teleost fishes, and 2 amphibians. For chronic toxicity, there were 15 CTVs with the corresponding water hardness for four species (two phyla and four families) from the literature and toxicity database shown in Table 2 and Table S2), which included a planktonic species, three vertebrate species including a cyprinid teleost fish, and two non-cyprinid teleost fishes. The CTVs for silver ranged from 0.12 μg/L to 50 μg/L, while the water hardness ranged from 27.5 mg/L to 340 mg/L as CaCO_3_. The toxicity data of silver to freshwater aquatic organisms are sufficient for ATVs and insufficient for CTVs according to the requirements of WQC derivation mentioned above. Therefore, CMC was derived via the SSD method and CCC was derived via the ACR method.

**Figure 1:**
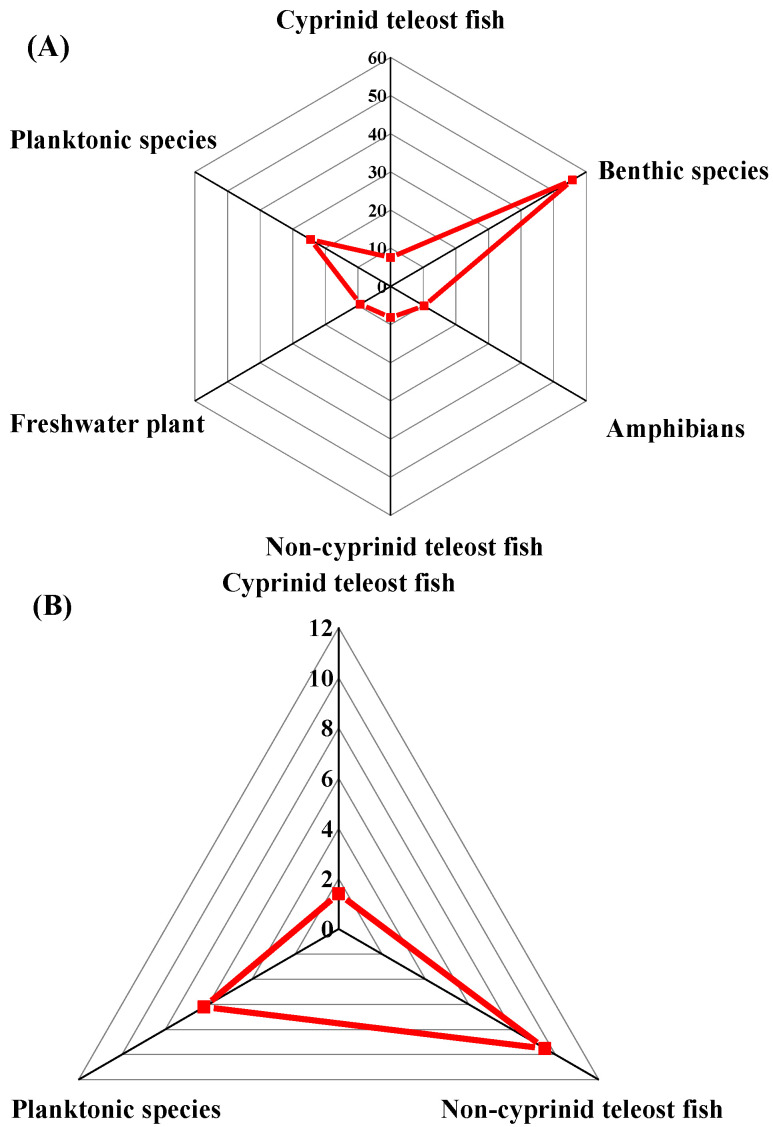
The geometric mean of (**A**) the SMAVs and (**B**) the SMCVs of silver to various taxonomic groups of freshwater aquatic organisms (concentrations in μg/L).

The 121 ATVs contained a high proportion of vertebrate species (61.16%), including cyprinid teleost fishes, non-cyprinid teleost fishes, and amphibians. The percentage of invertebrate species was 37.71%, including the planktonic species and benthic species, while the percentage of freshwater plants was 0.82% for ATVs of silver. The geometric mean of the SMAVs to silver in increasing order evidenced that: cyprinid teleost fishes < non-cyprinid teleost fishes < amphibians < freshwater plants < planktonic species < benthic species, for various taxonomic groups of freshwater aquatic organisms (Figure 1A). Meanwhile, the 15 CTVs included cyprinid teleost fishes (20.00%), non-cyprinid teleost fishes (26.67%), and planktonic species (53.33%). The geometric mean of the SMCVs to silver in increasing order evidenced that: cyprinid teleost fishes < planktonic species < non-cyprinid teleost fishes, for various taxonomic groups of freshwater aquatic organisms (Figure 1B). Overall, the cyprinid teleost fishes showed the most sensitivity to silver under both short-term and long-term exposure based on the SMAVs and SMCVs.

### 3.2. Derivation of the WQC for Silver

#### 3.2.1. Correlations between Water Hardness and Toxicity of Silver

**Figure 2:**
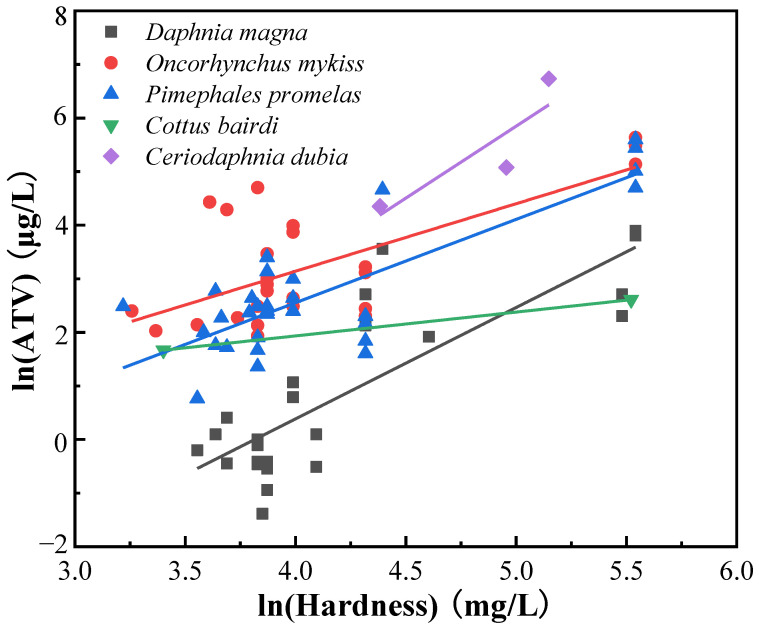
The linear relationship between the natural logarithms of the hardness and the natural logarithms of the ATVs of silver ions for *Daphnia magna*, *Oncorhynchus mykiss*, *Pimephales promelas*, *Cottus bairdi*, and *Ceriodaphnia dubia*.

Water hardness can affect the toxicity of silver to freshwater aquatic organisms. The least-squares regression of the natural logarithm of the ATVs was performed on the corresponding natural logarithm of the water hardness for all species, and significant correlations were obtained for *D. magna*, *O. mykiss*, and *Pimephales promelas* with *p* < 0.05 (Table S3). These results were similar to those in the silver WQC document issued by the USEPA, which found that the least-squares regression of the natural logarithms of the ATVs and the natural logarithms of hardness were statistically significantly correlated (*p* = 0.01) for *D. magna*, *O. mykiss*, and *P. promelas* [6]. This may be due to higher concentrations of Ca^2+^ and Mg^2+^, which can both repel silver ions through the competitive adsorption of biological cell membranes, thus reducing the acute toxicity of silver [38]. That is, silver is more toxic in soft water than in hard water for freshwater aquatic organisms over short-term exposure. According to the criteria guidelines issued by the USEPA and the MEEC, it is necessary to use a covariance analysis to take into account the relationship between the acute toxicity of silver and the hardness of water. A subset of the ATVs for five species (three fishes and two invertebrates) were used to calculate the *K*_pooled_, in which the highest hardness was at least three times the lowest and the highest hardness was at least 90 mg/L greater than the lowest one. In detail, the individual species slopes of the natural logarithms of the ATVs and the natural logarithms of hardness are 0.4445–2.6853 (*p* < 0.05) for *D. magna*, *O. mykiss*, *P. promelas*, *Cottus bairdi*, and *Ceriodaphnia dubia*, as shown in Table S3. The analysis of the covariance model term describing the similarity of the hardness slopes between individual species was not statistically significant, with a *p*-value of 0.053 (*p* > 0.05.), thus, the *K*_pooled_ could be acceptably calculated, which was statistically equivalent to a model with individual slopes for each species. The *K*_pooled_ obtained by the statistically significant correlations between the natural logarithms of the normalized ATVs and the natural logarithms of the normalized water hardness for all species is 1.58 (*p* < 0.05) for acute silver toxicity (Figure 2). However, the possible influence of hardness on chronic silver toxicity for freshwater aquatic organisms could not be found due to the lack of CTVs, as mentioned above.

#### 3.2.2. Hardness-Dependent WQC for Silver

The ATVs of silver for freshwater aquatic organisms were adjusted to the approximate water hardness in China using a *K*_pooled_ value of 1.58. The water area proportion of water hardness distribution to total surface water area was 42%, 34%, 11%, and 13% for hardnesses of <150 mg/L, 150–300 mg/L, 300–450 mg/L, and >450 mg/L, respectively, in China according to the results of the third China National Surface Water Quality Evaluation [28]. Based on the principle of the equal distribution of data, the ATVs were adjusted to five hardness levels by the hardness correction equation (Equation (1)) with the *K*_pooled_. After hardness adjustment, the ranked SMAVs_adj_ ranged from 0.66 μg/L to 14,400.63 μg/L with a hardness of 100 mg/L as CaCO_3_ (Table 1).

After the hardness adjustment of ATVs, based on the SMAVs_adj_, the three species most sensitive to silver were *Oryzias latipes*, *Lymnaea luteola*, and *Lebistes reticulatus*, two of which are kinds of fish. The three most tolerant species to silver, of the 35 species studied, were *Gammarus pseudolimnaeus*, *Philodina acuticornis*, and *Tanytarsus dissimilis*, which contained two kinds of benthic species. The results were similar to the sensitivity rank before hardness adjustment as mentioned above. In the silver WQC document issued by USEPA, the most sensitive species were *D. magna* and *P. promelas*, and the least sensitive species were *G. pseudolimnaeus* and *P. acuticornis* [6]. Therefore, teleost fish may be used as indicators of silver pollution in water for short-term exposure and benthic species may show the least sensitivity to silver in water. The *G. pseudolimnaeus* and *P. acuticornis* may be tolerant of silver ions.

**Table 1:** The ATV of silver for freshwater aquatic organisms in China, and the SMAV_adj_ ranked in order of sensitivity to silver for freshwater aquatic organisms with adjustment to a water hardness of 100 mg/L as CaCO_3_.

Rank	Species	Phyla	Families	*N*	Hardness (mg/L)	ATV (μg/L)	SMAV_adj_ (μg/L)
1	*Oryzias latipes*	Chordata	Adrianichthyidae	2	40	0.14–0.17	0.66
2	*Lymnaea luteola*	Mollusca	Lymnaea	1	195	4.2	1.46
3	*Lebistes reticulatus*	Chordata	Poeciliidae	1	250	6.44	1.51
4	*Duttaphrynus melanostictus*	Chordata	Bufonidae	1	185	4.1	1.55
5	*Puntius sophore*	Chordata	Cyprinidae	1	250	7.55	1.77
6	*Cyprinus carpio*	Chordata	Cyprinidae	1	118	3.8	2.92
7	*Moina dubia*	Arthropoda	Moinidae	1	109	4.5	3.93
8	*Daphnia magna*	Arthropoda	Daphniidae	24	35–255	0.25–49	4.27
9	*Channa punctatus*	Chordata	Channidae	1	250	18.89	4.42
10	*Hyalella azteca*	Arthropoda	Hyalellidae	2	35.2–47.8	1–1.9	5.66
11	*Tubifex tubifex*	Annelida	Tubificid	1	245	31	7.49
12	*Cyclops varicans*	Arthropoda	Cyclopidae	1	109	12	10.47
13	*Cottus bairdi*	Chordata	Cottidae	2	30–250	5.3–13.6	10.66
14	*Jordanella floridae*	Chordata	Istiophoridae	2	44.3–48	9.2–9.6	32.05
15	*Alona affinis*	Arthropoda	Chydoridae	1	109	37	32.28
16	*Monopterus albus*	Chordata	Synbranchidae	1	21	2.8	33.21
17	*Pimephales promelas*	Chordata	Cyprinidae	30	25–255	2.15–270	33.41
18	*Isonychia bicolor*	Arthropoda	Isonychiidae	1	35.2	6.8	35.58
19	*Ceriodaphnia reticulata*	Arthropoda	Daphniidae	1	45	11	38.99
20	*Daphnia pulex*	Arthropoda	Daphniidae	1	45	14	49.63
21	*Simocephalus vetulus*	Arthropoda	Daphniidae	1	45	15	53.17
22	*Oncorhynchus mykiss*	Chordata	Salmonidae	26	26–255	6.9–280	59.28
23	*Ictalurus punctatus*	Chordata	Ictaluridae	1	44.8	17.3	61.76
24	*Cambarus diogenes*	Arthropoda	Cambaridae	1	100	65.85	65.85
25	*Chironomus tentans*	Arthropoda	Chironomidae	1	25	10.4	93.58
26	*Gambusia affinis*	Chordata	Poeciliidae	1	35.2	23.5	122.94
27	*Lepomis macrochirus*	Chordata	Centrarchidae	3	35.2–44.7	13–64	128.30
28	*Ceriodaphnia dubia*	Arthropoda	Daphniidae	3	80–172	77.6–839.95	153.37
29	*Macrobrachium nipponense*	Arthropoda	Palaemonidae	1	104	170	159.76
30	*Scenedesmus dimorphus*	Chlorophyta	Scenedesmaceae	1	11.3	9.3	294.55
31	*Euphlyctis hexadactylus*	Chordata	Ranidae	1	20	25.7	329.35
32	*Aplexa hypnorum*	Mollusca	Physidae	2	44.7–50.4	83–241	460.72
33	*Tanytarsus dissimilis*	Arthropoda	Chironomidae	1	48	3200	10,240.45
34	*Philodina acuticornis*	Aschelminthes	Philodinidae	1	25	1400	12,597.16
35	*Gammarus pseudolimnaeus*	Arthropoda	Gammaridae	1	48	4500	14,400.63

*N* = The number of ATVs.

**Figure 3:**
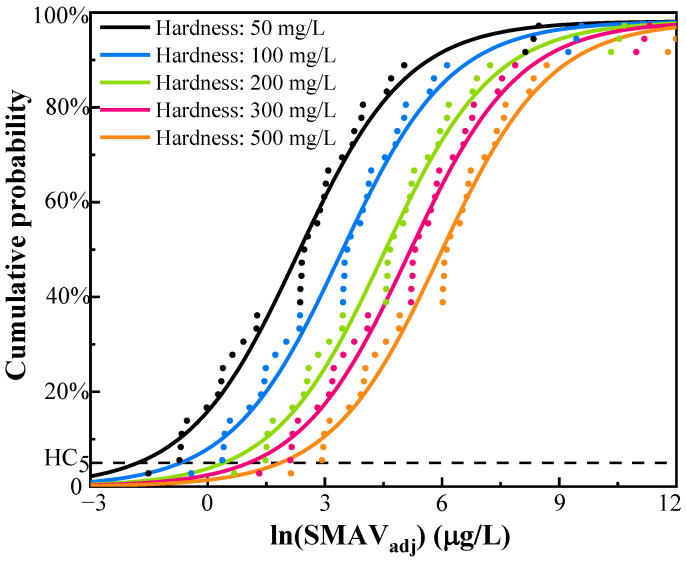
The SSD curves of silver ions for different hardness levels (50, 100, 200, 300, and 500 mg/L as CaCO_3_).

The SMAVs_adj_ of silver were employed to fit SSD curves and the Adj *R*^2^ of SSD models were 0.9797, 0.9714, 0.9403, 0.8675, and 0.7197, for the sigmoid model, Gompertz model, Lorentzian model, Logarithm model, and Exponential Growth model, respectively, with the SMAVs_adj_ as the independent variable and the cumulative probability of the species as the dependent variable (Table S4). Compared with other models, the sigmoid model exhibited the best fit for the SMAVs_adj_ of silver with the greatest Adj *R*^2^ value, 0.9797. Based on the sigmoid model, the SSD curves shifted from left to right and acute HC_5_ increased with the increasing hardness, indicating the acute toxicity of silver decreased when the hardness increased (Figure 3). The acute HC_5_ of silver calculated from sigmoid model was 0.17–6.42 μg/L in a hardness of 50–500 mg/L as CaCO_3_ (Table 3). Currently, most studies set the value of 2 as the assessment factor if the toxicity data cover at least three phyla and eight families [24,39,40]. The same assessment factor of 2 was applied in this criteria derivation to ensure the consistency of results [41]. The CMC of silver, using the acute HC_5_ divided by the assessment factor of 2, was 0.08–3.21 μg/L in a hardness of 50–500 mg/L as CaCO_3_ (Table 3). It was clear that the CMC at the lowest hardness (50 mg/L) was nearly 40 times less than that at the highest hardness (500 mg/L) in China, indicating the water hardness should be considered to derive the CMC for protecting the freshwater aquatic organisms from silver. The CMC can also be expressed with the equation $\mathrm{CMC}={\;e}^{1.58\ln\left(H_{{\mathrm{CaCO}}_{3}}\right)-8.68}$, with water hardness ($H_{{\mathrm{CaCO}}_{3}}$, mg/L) as an independent variable.

Considering the lack of CTVs, the ACR method was used to calculate the CCC based on the guidelines from both the USEPA and the MEEC [6,25,42]. The SACRs of *D. magna*, *O. mykiss*, and *P. promelas* were calculated as 0.75, 39.36, and 4.06, respectively, and the FACR values calculated as the geometric mean of each SACR was 4.92 (Table 2). As a result, the CCC derived from CMC and FACR was 0.02–0.65 μg/L in a hardness of 50–500 mg/L as CaCO3, shown in Table 3. The CCC can also be expressed with the equation $\mathrm{CCC}={\;e}^{1.58\ln\left(H_{{\mathrm{CaCO}}_{3}}\right)-10.28}$, with water hardness ($H_{{\mathrm{CaCO}}_{3}}$, mg/L) as an independent variable.

**Table 2:** The CTV of silver for freshwater aquatic organisms in China, and the SACRs for silver.

Species	*N*	Hardness (mg/L)	CTV (μg/L)	SACRs
*D. magna*	8	35–180	2.6–29	0.75
*O. mykiss*	3	27.5–37	0.12–12	39.36
*Oreochromis niloticus*	1	340	50	-
*P. promelas*	3	30.5–206	0.53–98	4.06

*N*: The number of CTV.

**Table 3:** The fitting evaluation results of the sigmoid model, and the values of the acute HC_5_, CMC, and CCC for different hardness levels.

Hardness (mg/L)	a	b	x_0_	Adj *R*^2^	HC_5_ (μg/L)	CMC (μg/L)	CCC (μg/L)
50	0.9819	1.4004	2.3066	0.9797	0.17	0.08	0.02
100	0.9819	1.4004	3.4051	0.9797	0.50	0.25	0.05
200	0.9819	1.4004	4.5036	0.9797	1.50	0.75	0.15
300	0.9819	1.4004	5.1461	0.9797	2.86	1.43	0.29
500	0.9819	1.4004	5.9557	0.9797	6.42	3.21	0.65

Sigmoid model: $y=a/\left(1+e^{-\frac{x-x_{0}}{b}}\right).$

### 3.3. The Comparison with Other WQC and Water Quality Standards for Silver

WQC for silver to protect freshwater aquatic organisms has been investigated and published by many countries including the United States, Canada, Australia, and New Zealand [6,14,15]. For example, the CMC of silver issued by the USEPA was ${\;e}^{1.72\ln\left(\mathrm{hardness}\right)-6.52}$, with water hardness as an independent variable, for protecting freshwater aquatic organisms [6]. The CMC for silver in the United States (4.1 μg/L) was 16.4-fold greater than the CMC (0.25 μg/L) in this study with a hardness of 100 mg/L as CaCO_3_ to protect freshwater aquatic organisms. The WQC should be derived from the toxicity data of native species to reduce the possible influence of natural history, water characteristics, specific taxonomic groups, types of habitats, and the geographical distribution of species in different ecosystems [15,24,25]. The different CMC in the United States and China may be attributed to the different characteristic biota and local species in the two countries. The *K*_pooled_ value of acute toxicity in the United States (1.72) and that in our study (1.58) were similar, although they were calculated from different individual species slopes involving different species. This suggests that the influence of hardness on the acute toxicity of silver is similar and acceptable, and the *K*_pooled_ in this study could strengthen the validity of the hardness adjustment. The CCC of silver was 0.12 μg/L according to the USEPA and 0.13 μg/L as reported by Diamond et al. (1990) for the New River of Virginia in the United States for protecting freshwater aquatic organisms [6,43]. The CCC of silver issued by Canada is 0.25 μg/L, and the high trigger values of silver issued by Australia and New Zealand are 0.05 μg/L. The maximum permissible addition of silver issued by the European Union is 0.082 μg/L [44]. These values were established with similar methods to our research for protecting freshwater aquatic organisms [14,15]. These guideline values for silver all fall within the range of the CCC values (0.02–0.65 μg/L) and hardness values (50–500 mg/L CaCO_3_) found in our study, and are even similar to the CCC in certain hardnesses.

Currently, the limits on silver are 50 μg/L for drinking water and 100 μg/L for the maximum allowable daily concentration of sewage in China [45,46]. Meanwhile, the concentration of silver in freshwater is in the range of 0.01–3.50 μg/L [30]. It is clear that some concentrations are apparently greater than the CMC and CCC predicted in this study, and the risk to the environment should be taken into account for silver in freshwater in China. The toxicity of metal ions for freshwater aquatic organisms might be influenced by water quality characteristics, such as hardness, pH, or temperature [26,47,48]. For silver, a quantitative relationship between water hardness and silver toxicity was obtained by our study, as well as that of the USEPA [6]. The hardness-dependent WQC of other metal ions have also been derived, such as cadmium and lead [47,48]. Additionally, other factors, such as dissolved organic carbon and chloride ions may influence the toxicity of silver via the formation of complexes [14]. The quantitative relationship between the toxicity of silver and other water quality parameters and elements could not be discussed due to the lack of toxicity data for freshwater organisms in China, which should be investigated in the future. Due to the lack of CTVs for silver, the derivation of CCC was based on the ACR method instead of the SSD method. Further research on the chronic toxicity of silver in freshwater aquatic organisms should be undertaken. The possible influences of both bioaccumulation and bioconcentration effects should be taken into consideration regarding further WQC for silver [30,49,50].

## 4. Conclusions

The toxicity database of silver for freshwater aquatic organisms involved 121 ATVs for 35 species (6 phyla and 27 families) and 15 CTVs for 4 species (2 phyla and 4 families). Teleost fish showed sensitivity to silver after both short-term and long-term exposure and thus may be used as indicators of silver pollution in freshwater. Significant correlations between the water hardness and acute toxicity of silver were obtained for *D. magna*, *O. mykiss*, and *P. promelas*, and the *K*_pooled_ was 1.58 for the acute toxicity of silver (n = 85, *p* < 0.05). The acute HC_5_ was calculated to be 0.17–6.42 μg/L at a hardness of 50–500 mg/L as CaCO_3_. The CMC and CCC can be expressed using the equation $\mathrm{CMC}={\;e}^{1.58\ln\left(H_{{\mathrm{CaCO}}_{3}}\right)-8.68}$, and $\mathrm{CCC}={\;e}^{1.58\ln\left(H_{{\mathrm{CaCO}}_{3}}\right)-10.28}$, respectively, with water hardness ($H_{{\mathrm{CaCO}}_{3}}$, mg/L) as an independent variable. The possible risk to the environment should be taken into account and given more concern regarding silver in freshwater in order to protect freshwater aquatic organisms.

## Data Availability

The data presented in this study are available on request from the corresponding author upon reasonable request.

## Supplementary Materials

The following supporting information can be downloaded at: https://www.mdpi.com/article/10.3390/ijerph19106067/s1. The supplementary tables (Tables S1–S4). Table S1. The ATVs of silver to freshwater aquatic organisms in China. Table S2. The CTVs of silver to freshwater aquatic organisms in China. Table S3. The individual species slopes and the *K*_pooled_ calculated for the ATVs vs. hardness relationship for silver. Table S4. The fitting evaluation results of the SSD models for silver.
