# Supplementary material for: Hardness-Dependent Water Quality Criteria for Protection of Freshwater Aquatic Organisms for Silver in China
Source: Int J Environ Res Public Health. 2022 May 17;19(10):6067. doi: 10.3390/ijerph19106067 (PMC9141652; doi:10.3390/ijerph19106067)
Supplement: Supplementary file 1 [file ijerph-19-06067-s001.zip › ijerph-1665952-supplementary.pdf]

Supplementary Material

1 Supplementary Table

Table S1. The ATVs of silver to freshwater aquatic organisms in China.

Table S2. The CTVs of silver to freshwater aquatic organisms in China.

Table S3. The individual species slopes and the K_{pooled} calculated for the ATVs vs. hardness relationship for silver.

Table S4. The fitting evaluation results of the SSD models for silver.

Table S1. The ATVs of silver to freshwater aquatic organisms in China.

Phyla	Families	Species	N	Hardness (mg/L)	ATV (μg/L)
Mollusca	Physidae	*Aplexa hypnorum*	2	44.7-50.4	83-241
Arthropoda	Chydoridae	*Alona affinis*	1	109	37
Arthropoda	Cambaridae	*Cambarus diogenes*	1	100	65.85
Arthropoda	Daphniidae	*Ceriodaphnia reticulata*	1	45	11
Arthropoda	Daphniidae	*Ceriodaphnia dubia*	3	80-172	77.6-839.95
Chordata	Channidae	*Channa punctatus*	1	250	18.89
Arthropoda	Chironomidae	*Chironomus tentans*	1	25	10.4
Chordata	Cottidae	*Cottus bairdi*	2	30-250	5.3-13.6
Chordata	Cyprinidae	*Cyprinus carpio*	1	118	3.8
Arthropoda	Cyclopidae	*Cyclops varicans*	1	109	12
Arthropoda	Daphniidae	*Daphnia magna*	24	35-255	0.25-49
Arthropoda	Daphniidae	*Daphnia pulex*	1	45	14
Chordata	Bufonidae	*Duttaphrynus melanostictus*	1	185	4.1
Chordata	Ranidae	*Euphlyctis hexadactylus*	1	20	25.7
Arthropoda	Gammaridae	*Gammarus pseudolimnaeus*	1	48	4500
Chordata	Poeciliidae	*Gambusia affinis*	1	35.2	23.5
Arthropoda	Hyalellidae	*Hyalella azteca*	2	35.2-47.8	1-1.9
Chordata	Ictaluridae	*Ictalurus punctatus*	1	44.8	17.3
Arthropoda	Isonychiidae	*Isonychia bicolor*	1	35.2	6.8

Chordata	Istiophoridae	*Jordanella floridae*	2	44.3-48	9.2-9.6
Mollusca	Lymnaea	*Lymnaea luteola*	1	195	4.2
Chordata	Poeciliidae	*Lebistes reticulatus*	1	250	6.44
Chordata	Centrarchidae	*Lepomis macrochirus*	3	35.2-44.7	13-64
Arthropoda	Palaemonidae	*Macrobrachium nipponense*	1	104	170
Arthropoda	Moinidae	*Moina dubia*	1	109	4.5
Chordata	Synbranchidae	*Monopterus albus*	1	21	2.8
Chordata	Salmonidae	*Oncorhynchus mykiss*	26	26-255	6.9-280
Chordata	Adrianichthyidae	*Oryzias latipes*	2	40	0.14-0.17
Chordata	Cyprinidae	*Puntius sophore*	1	250	7.55
Chordata	Cyprinidae	*Pimephales promelas*	30	25-255	2.15-270
Aschelminthes	Philodinidae	*Philodina acuticornis*	1	25	1400
Arthropoda	Daphniidae	*Simocephalus vetulus*	1	45	15
Chlorophyta	Scenedesmaceae	*Scenedesmus dimorphus*	1	11.3	9.3
Annelida	Tubificid	*Tubifex tubifex*	1	245	31
Arthropoda	Chironomidae	*Tanytarsus dissimilis*	1	48	3200

N：The number of ATVs

Table S2. The CTVs of silver to freshwater aquatic organisms in China.

Phyla	Families	Species	N	Hardness (mg/L)	CTV (μg/L)
Arthropoda	Daphnidae	*Daphnia magna*	8	35-180	2.6-29
Chordata	Salmonidae	*Oncorhynchus mykiss*	3	27.5-37	0.12-12
Chordata	Cichlidae	*Oreochromis niloticus*	1	340	50
Chordata	Cyprinidae	*Pimephales promelas*	3	30.5-206	0.53-98

N: The number of CTVs

Table S3. The individual species slopes and the K_{pooled} calculated for the ATVs vs. hardness relationship for silver.

Species	n	Slope	R^2
Daphnia magna	24	2.0784*	0.5834
Oncorhynchus mykiss	26	1.2596*	0.4647
Pimephales promelas	30	1.5552*	0.6530
Cottus bairdi	2	0.4445	-
Ceriodaphnia dubia	3	2.6853	0.7673
K_{pooled}	85	1.5848*#	0.5777

* Slope is significantly different than 0 ($P<0.05$)
# Individual species slopes not significantly different ($P=0.053$)

Table S4. The fitting evaluation results of the SSD models for silver.

Model	Equation	a	b	Adj R^2
Sigmoid	$y=\frac{a}{1+e^{-\frac{x-x_0}{b}}}$	0.9819	1.4004	0.9797
Gompertz	$y=a\times e^{-e^{-\frac{x-x_0}{b}}}$	1.0392	2.3501	0.9714
Lorentzian	$y=\frac{a}{1+(\frac{x-x_0}{b})^2}$	1.2146	3.2292	0.9403
Logarithm	$y=a\ln(x-x_0)$	0.3764	1.5663	0.8675
Exponential Growth	$y=ae^{bx}$	0.1928	0.1465	0.7197

a, b: the constant of equation
